# Neutrophil gelatinase-associated lipocalin as a biomarker for short-term outcomes among trauma patients: A single-center observational study

**DOI:** 10.1371/journal.pone.0251319

**Published:** 2021-05-10

**Authors:** Sakura Minami, Tomoki Doi, Takeru Abe, Ichiro Takeuchi

**Affiliations:** 1 Department of Emergency Medicine, Yokosuka Kyosai Hospital, Yokosuka, Japan; 2 Department of Intensive Care Medicine, Kanagawa Children’s Medical Center, Yokohama, Japan; 3 Department of Emergency Medicine, Yokohama City University School of Medicine, Yokohama, Japan; Heidelberg University Hospital, GERMANY

## Abstract

**Background:**

Urinary biomarkers for organ dysfunction could predict the outcomes of severe trauma patients. However, the use of neutrophil gelatinase-associated lipocalin (NGAL) as a biomarker of trauma is not well studied.

**Objectives:**

To evaluate the association between the short-term prognosis of trauma patients and NGAL levels.

**Methods:**

We conducted a single center study and compared predictive performances between NGAL levels and the trauma severity.

**Results:**

A total of 104 patients were included in the study. Patients were divided into two groups based on ISS score of 16. There was no significant difference in patient characteristics based on trauma severity. However, the lactate level was significantly higher in the more severe group. There was a significant association between urinary NGAL levels and trauma severity indicators, such as intensive care unit stay (ICU) (p = 0.005) and emergency care unit (ECU) stay (p = 0.049). In addition, receiver operating curve analysis showed that as a predictor, NGAL could be used for detecting severity with moderate precision, especially for short-term outcomes (specificity 70.6 for ICU and 69.0 for ECU stay).

**Conclusion:**

In this study, we revealed that the level of NGAL could predict the degree of invasiveness in trauma patients with moderate precision and estimate the duration of treatment during the acute phase. It is necessary to examine the validity of the findings of this study using a prospective, cohort, and multi-center collaborative study design.

## Introduction

To this date, trauma treatment is still difficult and challenging, and early severity prediction is important for improving prognosis in trauma patients [1]. Fast evaluation could lead to early diagnosis and treatment and subsequently reduce CT time and mortality [2]. For assessing the severity of the trauma, physiological indicators, such as Revised Trauma Score (RTS) and anatomical indicators, such as Injury Severity Score (ISS) are used with validated accuracy for prediction, but most of them can be utilized only retrospectively [3–5]. It is difficult to predict the severity based on the information available in the initial stage of treatment, especially at first contact with a patient in an emergency room.

Urinary biomarkers for organ dysfunction could predict outcomes of severe trauma patients, such as those involved in battle fields [6]. Among them, neutrophil gelatinase-associated lipocalin (NGAL) is a useful biomarker for organ damage in the human body [7–9], and elevated NGAL is associated with renal failure and heart failure [10–13]. However, an association between NGAL levels and the outcome of less severe trauma patients in the emergency department of general hospitals is unknown. It would be beneficial to use a biomarker for rapid trauma evaluation, so that an institute can prepare and provide a patient with appropriate resources. Thus, in this study, we examined the association between the short- and long-term prognosis of trauma patients and NGAL as a biomarker of trauma. The significant association among them could contribute to the early determination of trauma severity and treatment plans based on the level of NGAL.

## Materials and methods

This is a single center study at the Yokosuka Kyosai Hospital, Japan. The study was carried out for a year, between October 1, 2017 and September 30, 2018. The facility has a critical care center with a 740-bed hospital in Yokosuka City which has a population of 395,903 [14]. This study was conducted with the approval of the institutional review board at the Yokosuka Kyosai Hospital (The Ethics Committee No. 18–14). The need for consent was waived by the ethics committee.

We obtained patient characteristics and clinical information from the visiting hospital, such as age in years, sex, lactate level, creatinine at admission and at day 5, ISS score, previous history of chronic kidney disease, CKD, and heart disease. Further we chose large blood transfusion, surgery, and transcatheter arterial embolization (TAE) as trauma-specific treatment. The primary outcomes were intensive care unit (ICU) stay period, the emergency care unit (ECU) stay period, and length of hospital stay (LOS) because no mortality was observed during the study period. Additionally, urinary NGAL was measured at the time of visit of the patient as a routine blood test procedure and reexamined the day after admission. We measured NGAL with the CLIA (chemiluminescent immunoassay) technique, using the ARCHITECT uNGAL assay from the Abbott Japan, Tokyo, Japan. We did not utilize creatinine correction to urinary NGAL, because original values and corrected ones are highly correlated and a few previous studies have used the former values [15]. Inclusion criteria included trauma patients who were transferred by an ambulance or direct visit, and subsequently hospitalized in the institution during the study period. Exclusion criteria included those with missing information above, with traumatic cardio-pulmonary arrest, CPA, or those under 15 years of age.

Univariate analysis was performed on the items according to the severity of trauma (ISS cutoff value = 16). The Mann-Whitney U test was used for continuous variables and Fisher’s exact test for categorical variables. Furthermore, in order to examine the relationship between the outcome and NGAL levels, multiple regression analysis was performed using all measurement items. Collinearity was checked with the variation inflation factor (VIF). The VIF value larger than four was considered to violate a premise of collinearity and to cast multi-collinearity which determined if a variable should be excluded from the model [16]. In addition, we compared predictive performances between NGAL levels and ISS. Specifically, we used receiver operating curve (ROC) analysis and obtained each the area under the curve (AUC) its 95% confidence interval, sensitivity, specificity, positive predictive value, negative predictive value, and a cut-off value, in which outcomes were dichotomized using the median for a cut-off value: 1 day for ICU stay, 3 days for ECU stay, and 16 days for LOS. We utilized the DeLong Method for comparing two AUCs [17]. A two-sided, *p* < 0.05 was considered statistically significant. For statistical analysis software, IBM-SPSS Statistics 23 (IBM, Chicago, USA) and MedCalc for Windows, version 15.0 (MedCalc Software, Ostend, Belgium) were used.

## Results

There were 246 trauma patients who visited the institution and were hospitalized during the study period. After the exclusion criteria were applied, a total of 104 patients were included for further analysis. There was no significant difference in patient characteristics based on trauma severity, except that lactate level was significantly high in the more severe group with ISS ≥16 (p = 0.006; Table 1). More massive transfusions and TAE were performed with the more severe group than the less severe group (p = 0.045 and 0.004, respectively). The more severe group had significantly longer stay periods than the less severe group in ICU (3 vs. 1 day), ECU (5 vs. 3 days), and LOS (31 vs. 8 days) (in all p < 0.001). We observed one acute kidney injury, AKI, patient only in ISS <16 group, but none in ISS > = 16 group. There were no significant differences in creatinine at admission and day 5, nor time course change among them (Table 1).

**Table 1:** Outcomes and patient characteristics based on trauma severity.

	ISS < 16 median (interquartile range)/ frequency (%)		ISS > = 16 median (interquartile range)/ frequency (%)		p value
Age	51	(35–73)	60	(43–74)	0.392
Female	21	(31%)	13	(37%)	0.659
NGAL	10.0	(10.0–23.8)	10.0	(10.0–31.8)	0.786
Creatinine at admission	0.78	(0.64–0.98)	0.85	(0.73–1.03)	0.184
Creatinine day 5	0.69	(0.52–0.87)	0.70	(0.55–0.86)	0.988
Lactate level	1.8	(1.2–2.7)	2.5	(1.9–4.8)	0.006
Previous history of chronic kidney disease	2	(3%)	2	(6%)	0.608
Previous history of cardiovascular disease	21	(32%)	8	(23%)	0.368
Massive transfusion	4	(6%)	7	(20%)	0.045
Emergency surgery	11	(17%)	11	(31%)	0.127
TAE	0	(0%)	5	(14%)	0.004
ICU stay (days)	1	(0–2)	3	(1–4)	< 0.001
HCU stay (days)	3	(2–4)	5	(3–8)	< 0.001
Length of stay, LOS (days)	8	(2–26)	31	(21–41)	< 0.001

NGAL: neutrophil gelatinase-associated lipocalin; ISS: Injury Severity Score; TAE: transcatheter arterial embolization; ICU: intensive care unit; HCU: high-care unit; SE: standard error

Table 2 showed that, in multivariate analysis, NGAL level was a significant and independent factor for predicting the length of ICU stay and ECU stay (p = 0.005 and 0.049, respectively). Lactate level and ISS were also significant factors for all of the outcomes, including LOS (p < 0.01 in all). All multivariate models were able to detect a large effect size, in which adjusted regression coefficients, or R-squared value were greater than 0.26 with more than 55 sample size [18, 19].

**Table 2:** Factors and NGAL related to outcomes.

	ICU stay				HCU stay				Length of stay			
	B	SE	β	p-value	B	SE	β	p-value	B	SE	β	p-value
Female	-0.045	0.18	-0.028	0.804	-0.115	0.139	-0.075	0.410	-0.219	0.289	-0.078	0.450
Age	0.004	0.004	0.111	0.399	-2.90*10^−5^	0.003	-0.001	0.993	-0.008	0.007	-0.129	0.282
NGAL	0.090	0.08	0.138	0.266	0.152	0.059	0.242	0.012	0.3	0.127	0.261	0.020
Creatinine at admission	0.459	0.238	0.243	0.059	0.451	0.198	0.231	0.025	-0.149	0.421	-0.041	0.724
Lactate level	0.042	0.022	0.222	0.059	0.039	0.019	0.194	0.039	0.073	0.040	0.195	0.072
ISS	0.465	0.152	0.310	0.003	0.570	0.125	0.377	<0.001	1.16	0.261	0.417	<0.001
Renal disease	-0.625	0.361	-0.19	0.088	-0.488	0.325	-0.134	0.137	0.628	0.661	0.098	0.345
Cardiovascular disease	0.034	0.215	0.021	0.873	0.070	0.160	0.044	0.662	0.222	0.353	0.075	0.531
R^2^ (adjusted R^2^)	0.38	(0.31)			0.44	(0.39)			0.32	(0.26)		

B: regression coefficient; SE: standard error; β: standardized regression coefficient; NGAL: neutrophil gelatinase-associated lipocalin; ISS: Injury Severity Score; ICU: intensive care unit; HCU: high-care unit

**Fig 1:**
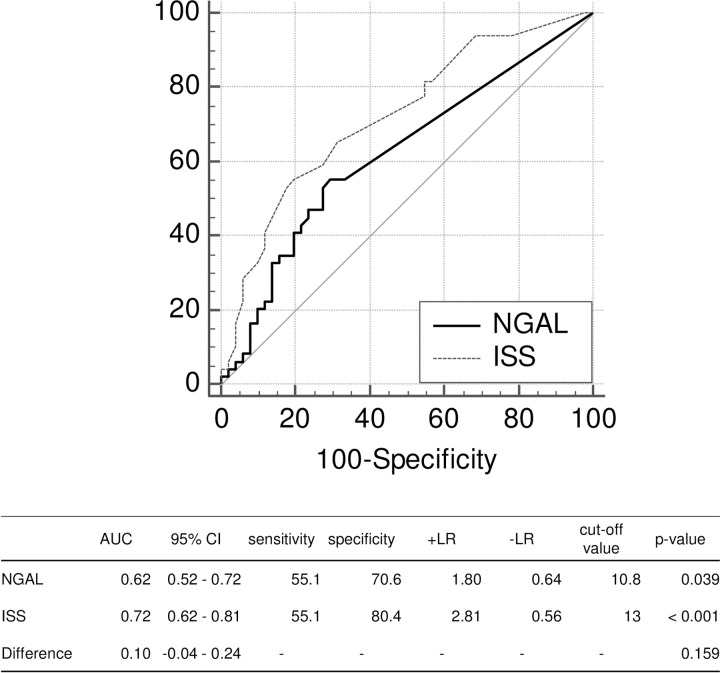
Comparison of the areas under the curve between NGAL levels and ISS on ICU stay. AUC: area under the curve; +LR: positive likelihood ratio; -LR: negative likelihood ratio; NGAL: neutrophil gelatinase-associated lipocalin; ISS: Injury Severity Score; ICU: Intensive Care Unit.

**Fig 2:**
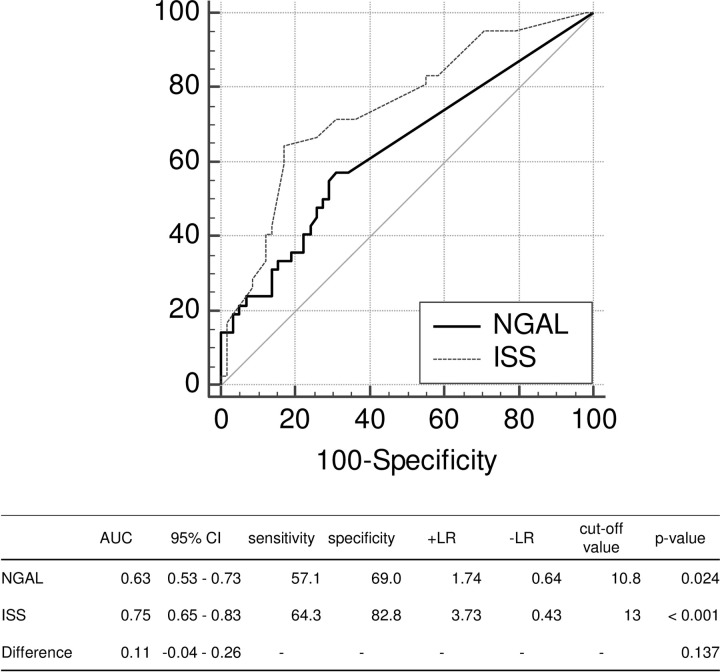
Comparison of the areas under the curve between NGAL levels and ISS on ECU stay. AUC: area under the curve; +LR: positive likelihood ratio; -LR: negative likelihood ratio; NGAL: neutrophil gelatinase-associated lipocalin; ISS: Injury Severity Score; ECU: Emergency Care Unit.

**Fig 3:**
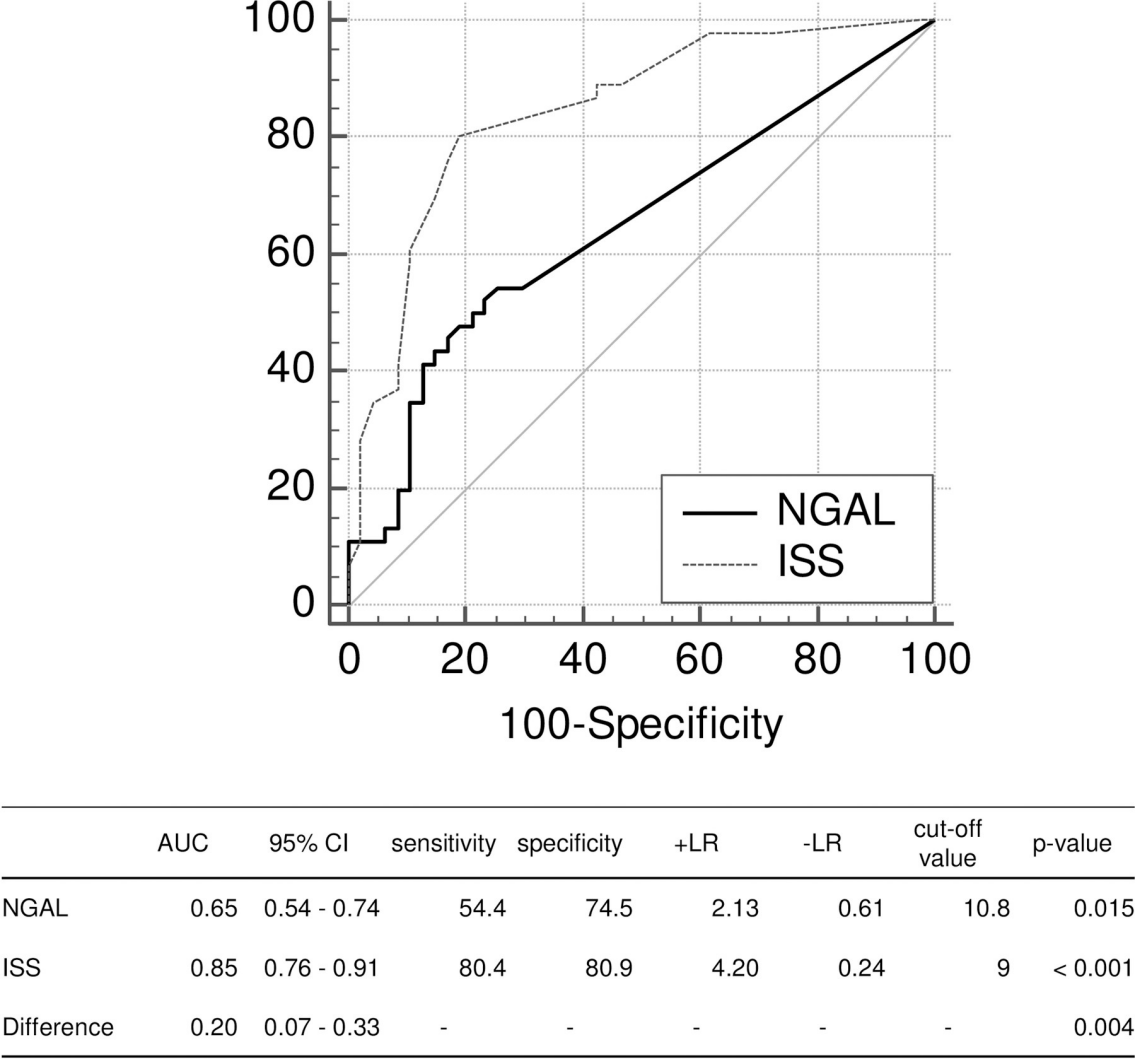
Comparison of the areas under the curve between NGAL levels and ISS on LOS. AUC: area under the curve; +LR: positive likelihood ratio; -LR: negative likelihood ratio; NGAL: neutrophil gelatinase-associated lipocalin; ISS: Injury Severity Score; LOS: length of hospital stay.

Regarding ICU and EICU stays, both NGAL levels and ISS were moderate predictors with high specificity (Figs 1 and 2). In addition, there was no difference in predicting performances between NGAL levels and ISS. As for LOS, although both obtained high specificity, ISS prediction was significantly better than NGAL (p = 0.004) (Fig 3).

## Discussion

In this study, we examined the association between NGAL as a biomarker for prognosis in trauma patients admitted to the emergency center. Our analysis showed that levels of urinary NGAL had a significant association with the trauma severity, ICU stay, ECU stay, and LOS. In addition, ROC analysis showed that as a predictor, NGAL levels could be used for detecting patient’s severity with moderate precision, especially for the short-term outcomes, such as ICU and ECU stays. In prior studies, it has been shown that NGAL could be used as a biomarker for endogenous diseases such as AKI and heart failure [10–13], and as one of the indicators of early intervention [7–9]. Additionally, our findings have confirmed the usefulness of NGAL in trauma patients. These results suggest that NGAL levels can independently predict the severity and short-term prognosis of trauma patients. Thus, the level of NGAL can serve as an indicator for clinicians to determine the immediate medical needs of trauma patients.

In addition to urinary NGAL, ISS and lactate levels were also significantly and independently related to the outcomes. Naturally, lower severity would be related to the shortened ICU stay period and ECU stay period because the study participants included minor cases as the target patients. Additionally, NGAL levels might be an independent predictor of trauma-severity scales since it is associated with diseases other than renal disorders such as heart failure and stroke. Changes in NGAL levels result from a defense mechanism by complex formation even in inflammatory diseases such as infections [10–13]. NGAL, a protein with a molecular weight of about 25 k Da, is; normally expressed very weakly in various human tissues, such as kidney, lung, stomach, and intestine; rapidly expressed in renal epithelial cells due to ischemia-reflux injury; then discharged in large amounts into urine [20]. It would be useful as a biomarker for early recognition of pathological changes. Therefore, our results suggest that a similar mechanism may act as a mediator even in an aggressive pathological condition such as trauma. Although the original severity index was often used as a measure for the severity of the trauma, we found that levels of NGAL can be utilized as a trauma severity index for short-term treatment. Measuring the level of NGAL in urine during an acute phase of trauma could predict a degree of invasiveness of the trauma and help in determining the treatment period for the acute phase.

### Limitations

This study has several limitations. First, the study was a single-center study. A small number of cases with less severe patients were included, and this selection bias might affect our results with only a moderate precision of NGAL on trauma-severity. Our study population could have been contained less severe trauma patients, and more severe patients might have been transported to a regional trauma center. Thus, a cation has been practiced to generalize the findings into other institute. Second, the multivariate models could be vulnerable to unmeasured confounders and it may be difficult to obtain a cause-effect relationship between NGAL levels and trauma severity. More severe patients might represent other indicators rather than NGAL, and it might be a result of these other indicators. However, our model shows that other severity index, such as ISS and lactate levels, were independently and significantly associated with the outcomes. Thus, a significant association between NGAL levels and trauma severity could still be established. Third, although NGAL could be a moderate predictor for short-term outcomes, trauma severity would still be the better predictor for long-term outcomes such as LOS. The levels of NGAL might represent only an immediate impact on the patient so that this one-point measurement can hardly explain the long-term effect of trauma. In addition, the severity measured by ISS could only be determined after a certain time has passed that lacks immediacy. In future studies measuring NGAL sequentially and analyzing dynamics is necessary to overcome this limitation. Fourth, the sites of trauma might affect the level of NGAL, and this was not included in our study. Although previous history such as CKD was considered in the study, further study might be needed to investigate more details in the anatomical characteristics of trauma. Fifth, the time between the injury and the hospital admission and blood test might vary among cases, which may cause an information and classification bias. A future study should include more accurate information on injury time. Sixth, an incidence of AKI could be higher among trauma population than the study population (1.5%). Urinary NGAL could differentiate AKI and no AKI within the high resource utilization cohort, indicating that it could be a confounder if using NGAL to indicate injury severity [21–24]. Thus, a generalizability of the study findings should be limited for a population with higher AKI rate in a utility of urinary NGAL. Lastly, this study was conducted only retrospectively. A prospective study with the multi-center design is needed to overcome various biases, including those mentioned above.

## Conclusions

In this study, we revealed that the level of NGAL could moderately predict the degree of invasiveness in trauma patients and estimate the duration of treatment during the acute phase. It is necessary to examine the validity of the findings of this study by a prospective, cohort, and multi-center collaborative study design.

## Supporting information

S1 Data (XLSX)
